# Bavachin and Corylifol A Improve Muscle Atrophy by Enhancing Mitochondria Quality Control in Type 2 Diabetic Mice

**DOI:** 10.3390/antiox12010137

**Published:** 2023-01-06

**Authors:** Myeong-Hoon Yeon, Eunhui Seo, Jong-Han Lee, Hee-Sook Jun

**Affiliations:** 1 College of Pharmacy and Gachon Institute of Pharmaceutical Sciences, Gachon University, Incheon 21936, Republic of Korea; 2 Lee Gil Ya Cancer and Diabetes Institute, Gachon University, Incheon 21999, Republic of Korea; 3 Department of Marine Bio and Medical Science, Hanseo University, Seosan-si 31962, Republic of Korea; 4 Gachon Medical Research Institute, Gil Hospital, Incheon 21565, Republic of Korea

**Keywords:** *Psoralea corylifolia*, muscle atrophy, type 2 diabetes, mitochondrial biogenesis, mitochondrial dynamics, mitophagy

## Abstract

Type 2 diabetes reduces muscle mass and function. Chronic inflammation and mitochondrial dysfunction play critical roles in muscle atrophy pathogenesis. Here, we investigated the effects of bavachin and corylifol A from *Psoralea corylifolia* L. seeds on muscle atrophy in dexamethasone-treated mice and in db/db mice. Bavachin and corylifol A enhanced muscle strength and muscle mass in dexamethasone-treated mice. In diabetic mice, they enhanced muscle strength and cross-sectional areas. Bavachin and corylifol A suppressed inflammatory cytokine (interleukin-6 and tumor necrosis factor-α) expression levels by downregulating nuclear factor-κB phosphorylation. They decreased the muscle atrophic factor (myostatin, atrogin-1, and muscle RING finger-1) expression levels. They activated the AKT synthetic signaling pathway and induced a switch from fast-type glycolytic fibers (type 2B) to slow-type oxidative fibers (types I and 2A). They increased mitochondrial biogenesis and dynamic factor (optic atrophy-1, mitofusin-1/2, fission, mitochondrial 1, and dynamin 1-like) expression levels via the AMP-activated protein kinase–peroxisome proliferator-activated receptor gamma coactivator 1-alpha signaling pathway. They also improved mitochondrial quality by upregulating the mitophagy factor (p62, parkin, PTEN-induced kinase-1, and BCL2-interacting protein-3) expression levels. Therefore, bavachin and corylifol A exert potential therapeutic effects on muscle atrophy by suppressing inflammation and improving mitochondrial function.

## 1. Introduction

More than 500 million people worldwide suffer from diabetes. Type 2 diabetes (T2D) accounts for >90% of all diabetes cases, and its incidence has increased significantly over the last 20 years [1]. It is a complex metabolic disease characterized by insulin resistance, hyperglycemia, and hyperlipidemia. Loss of muscle mass and function are observed in patients with T2D [2]. Decreased muscle function negatively affects muscle strength and aerobic capacity, which further increases the disability and mortality rates in patients with T2D [3]. Chronic low inflammation is a characteristic feature of T2D [4,5]. It promotes muscle atrophy by decreasing muscle protein synthesis and increasing ubiquitin–proteasome, lysosome–proteasome, and caspase 3-mediated proteolysis [6,7]. Increased expression of circulating tumor necrosis factor (TNF)-α in T2D induces aberrant nuclear factor (NF)-κB activity, thereby promoting reactive oxygen species (ROS) production [8]. Moreover, increased inflammation promotes protein degradation in T2D by upregulating the expression levels of atrogin-1 and muscle RING finger-1 (MuRF1), which are major factors of the ubiquitin–proteasome pathway in muscles [9].

Mitochondria have a complex interconnected reticulum and are maintained by remodeling processes, collectively known as mitochondrial quality control mechanisms [10]. Mitochondrial biogenesis, fission, fusion, and mitophagy are key events in mitochondrial quality control [10]. Peroxisome proliferator-activated receptor gamma coactivator 1-alpha (PGC-1α) is an expert regulator of mitochondrial biogenesis; its expression is induced by exercise and chemical activation of AMP-activated protein kinase (AMPK) in the skeletal muscle [11]. During biogenesis, fission/fusion allows mitochondria to divide, which leads to the proper organization of the mitochondrial network [12]. Skeletal muscle fibers are generally classified as slow fiber muscles (type 1) and fast fiber muscles (type 2) according to their mitochondrial content [13]. In patients with T2D, the proportion of type 1 fibers decreases but that of type 2X fibers increases, and the capacity for oxidative metabolism in skeletal muscle decreases [14]. Mitochondrial dysfunction is a common cellular event in the pathogenesis of T2D and is the main contributor to increased oxidative stress, apoptosis, and cell death [15,16]. Thus, the maintenance of mitochondrial function may be a potential therapeutic strategy for muscle atrophy treatment.

*Psoralea corylifolia* L. seed (PCS) is used as a traditional medicine in Korea. Its extract consists of several phytochemical components: bakuchiol, psoralen, isoflavone, bavachromene, isobavachromene, bavachalcone, isobavachalcone, corylifol A, and bavachinin [17,18]. PCS extracts exert antihyperglycemic effects, such as increased plasma insulin levels and reduced blood glucose and plasma cholesterol levels, in rats with T2D [19]. In addition, PCS extracts exert various physiological effects, including anti-inflammatory, antibacterial, antitumor, and anti-osteoporotic effects [20,21,22,23,24]. We previously reported that the PCS extract attenuated muscle atrophy in dexamethasone (DEX)-induced muscle atrophy models by suppressing oxidative stress and inflammation [25]. Two compounds, bavachin and corylifol A, isolated from PCS, have myogenic activity, as evidenced by the increase in the expression of MyoD in vitro [26,27]. In the present study, we explored the components of PCS that can enhance mitochondrial function and investigated their effects on muscle atrophy in diabetic mice. In addition, we elucidated the mechanism by which they regulated the mitochondrial quality.

## 2. Materials and Methods

### 2.1. Animals

All of the animal experiments were conducted in accordance with the ethical requirements of the Laboratory Animal Research Center at the College of Pharmacy, Gachon University. The experimental protocol was approved by the Gachon University Institutional Animal Care and Use Committee (GIACUC-R2018012). Seven week old C57BL/6N male mice were obtained from Orient Bio (Seongnam-si, Kyunggido, Republic of Korea) and allowed to adapt for one week before the study. C57BL/6N male mice (eight week old) were randomly divided into four groups: CON (control), DEX, DEX + bavachin (10 mg/kg/day), and DEX + corylifol A (10 mg/kg/day) groups. Muscle atrophy was induced in C57BL/6N male mice via an intraperitoneal injection of DEX (20 mg/kg/day for 10 d), and bavachin and corylifol A were orally administered for 12 d, starting two days before DEX administration. DEX (D1756; Sigma-Aldrich, St. Louis, MO, USA), bavachin (A1101; KOCbio, Deajun, Republic of Korea), and corylifol A (A1101; KOCbio) were dissolved in 9% Kolliphor HS 15 (Solutol; 42966; Sigma-Aldrich) and 10% dimethyl sulfoxide (DMSO; D1370; Duchefa Biochemie, Haarlem, NL). Grip strength was measured on day 12 prior to sacrifice. Six week old male non-diabetic, heterozygous C57BLKS/J-m/m mice and homozygous C57BLKS/J-db/db mice were purchased from Orient Bio. Twelve week old mice were divided into four groups: control + vehicle, db/db + vehicle, db/db + 10 mg/kg bavachin, and db/db + 10 mg/kg corylifol A groups. Control + vehicle and db/db + vehicle groups orally received 9% solutol + 10% DMSO. The db/db + bavachin group was orally administered 10 mg/kg bavachin dissolved in 9% solutol + 10% DMSO, and the db/db + corylifol A group was orally administered 10 mg/kg corylifol A dissolved in 9% solutol + 10% DMSO once daily for 40 d. Grip strength was determined on day 39 to measure muscle force before sacrifice.

### 2.2. Measurement of Grip Strength

One day before sacrifice, hindlimb muscle strength was measured using a grip strength meter (BIO-G53; BIOSEB, Pinellas Park, FL, USA), as described in our previous report [28]. Grip strength was calculated as force divided by final body weight (N/g).

### 2.3. Tissue Collection

After sacrificing the mice, muscle tissues were collected and weighed. The soleus (SOL), gastrocnemius (GA), extensor digitorum longus (EDL), quadriceps femoris (QD), and tibialis anterior (TA) muscles were isolated and stored at −80 °C until use.

### 2.4. Histological Analysis

GA muscles were immersed in an optimal cutting temperature (OCT) solution immediately after dissection and frozen at −80 °C. OCT blocks were cut to a thickness of 10 μm and stained with hematoxylin (30002; Muto Pure Chemicals Co., Ltd., Tokyo, Japan) and eosin (H&E, HT110132; Sigma-Aldrich). Subsequently, these stained sections were examined (200× magnification) for cross-sectional area (CSA) analysis under a confocal microscope (Nikon Intensilight C-HGFI, Tokyo, Japan) and NIS-element AR 4.00.00 software. The CSA of myofibers was then measured using the ImageJ software.

### 2.5. Multicolor Immunofluorescent Staining

Tissue sections (15 μm thickness) from OCT blocks were fixed in 10% neutral-buffered formalin for 20 min at room temperature and washed in phosphate-buffered saline (PBS). Subsequently, the fixed tissues were permeabilized in PBS containing 0.2% Triton X-100 at 25 °C for 40 min and incubated at 25 °C with a protein blocking solution (Dako, Santa Clara, CA, USA) for 1 h. For characterization of the skeletal muscle fiber, the sections were incubated overnight at 4 °C in a mixture of BA-F8, SC-71, and BF-F3 antibodies at a ratio of 1:100 (Developmental Studies Hybridoma Bank [DSHB], Houston, TX, USA): anti-MyHC I (BA-F8, mouse IgG2b), anti-MyHC 2A (SC-71, mouse IgG1), and anti-MyHC 2B (BF-F3, mouse IgM). The sections were subsequently incubated with the following secondary antibodies at 25 °C for 1 h: Alexa Fluor 555-labeled goat anti-mouse IgM (for BF-F3), DyLight 405 rabbit anti-mouse IgG2B (for BA-F8), and Alexa Fluor 488-labeled goat anti-mouse IgG1 (for SC-71). Fluorescent images were taken under an A1 plus confocal laser scanning microscope (A1 plus; Nikon, Tokyo, Japan) at 600× magnification. Fibers labeled blue, green, and red were considered to be of types I, 2A, and 2B fibers, respectively. The myofiber CSA was analyzed using ImageJ software. For fiber type analysis, we measured the CSA of at least 300 myofibers per section from six mice per group. This measurement covers at least 70% of all fibers within a muscle/cross-section.

### 2.6. Measurement of ROS Levels

Dihydroethidium (DHE) is a commonly used ethidium-based, redox-sensitive fluorescent probe. To determine ROS levels, muscle sections were incubated with DHE (D7008; Sigma-Aldrich, St. Louis, MO, USA) for 30 min at 37 °C. DHE staining was quantified using fluorescence microscopy by measuring pixels exceeding a specified threshold set to remove any interference from the background fluorescence. The entire cross-sectional area was used for quantification, and the percentage of areas with positive staining was calculated.

### 2.7. Western Blotting Analysis

GA muscles were homogenized in the mammalian protein extract buffer (78501; GE Healthcare Life Sciences, Chicago, IL, USA) containing a protease inhibitor mixture (P8340; Sigma-Aldrich) and phosphatase inhibitors (P5726 and P0044; Sigma-Aldrich). Equal amounts of protein were separated by sodium dodecyl sulfate-polyacrylamide gel electrophoresis followed by transfer to membranes. Membranes were blocked in a blocking solution containing 5% skim milk or 5% bovine serum albumin for 1 h, and incubated overnight with the following primary antibodies: anti-4-hydroxynonenal (4HNE; MAB3249; RND Systems, Minneapolis, MN, USA), anti-p70S6K (#9202; Cell Signaling, Danvers, MA, USA), anti-p-p70S6K (#9206; Cell Signaling), anti-4E-BP1 (#9452; Cell Signaling), anti-p-4E-BP1 (#2855; Cell Signaling), anti-mammalian target of rapamycin (anti-mTOR; #2972; Cell Signaling), anti-p-mTOR (#2971; Cell Signaling), anti-PGC-1α (ab54481, Abcam, Cambridge, UK), anti-nuclear respiratory factor 1 (NRF1; #46743, Cell Signaling), anti-interleukin (IL)-6 (ab9324; Abcam), anti-TNFα (SC-133192; Santa Cruz biotechnology, Dallas, TX, USA), anti-NF-kB (#8242, Cell Signaling), anti-p-NF-kB (#3033, Cell Signaling), anti-AMPKα (#2532, Cell Signaling), anti-p-AMPKα (#2535, Cell Signaling), anti-p62 (#5114; Cell Signaling), anti-mitofusin 1 (MFN1; 13798-1-AP; Proteintech, Rosemont, IL, USA), anti-MFN2 (#9482; Cell Signaling), anti-OPA1 (#612606; BD Biosciences, Franklin Lakes, NJ, USA), anti-fission 1 (FIS1; 10956-1-AP; Proteintech), anti-dynamin 1-like (DNM1L, also known as DRP1; #8570; Cell Signaling), anti-LC3β (#2775; Cell Signaling), anti-Parkin (#4211; Cell Signaling), anti-PTEN-induced kinase-1 (PINK1; sc-517353; Santa Cruz Biotechnology), anti-BCL2-interacting protein-3 (BNIP3; ab109362; Abcam), anti-MuRF1 (ab172479; Abcam), anti-muscle atrophy F-box (MAFbx; atrogin-1; sc-166806; Santa Cruz Biotechnology), anti-GDF8/myostatin (ab203076; Abcam), anti-mitochondrial transcription factor A (TFAM; sc-166965; Santa Cruz Biotechnology), anti-MyHC 1 (BA-F8; DSHB), anti-MyHC 2B (BF-F3; DSHB), anti-troponin I-FS (TnI FS; sc-514969; Santa Cruz Biotechnology), anti-troponin I-SS (sc-514899; Santa Cruz biotechnology), and anti-glyceraldehyde 3-phosphate dehydrogenase (GAPDH; sc-32233; Santa Cruz biotechnology). The membrane was washed three times for 10 min with Tris-buffered saline with Tween-20, followed by incubation with horseradish peroxidase-conjugated goat anti-rabbit IgG or goat anti-mouse IgG secondary antibodies. The target complex was detected using the Chemidoc XRS+ system with Image Lab software (Bio-Rad, Hercules, CA, USA), and quantified using the Image Lab program.

### 2.8. Transmission Electron Microscopy (TEM) Analysis

After sacrifice, each GA muscle piece (1 × 1 × 1 mm) was fixed in 2.5% glutaraldehyde and 1% osmium tetroxide. The samples were dehydrated in graded ethanol, and infiltrated with propylene oxide before being embedded with epoxy resin (Poly bed 812 kit; Polysciences, Inc., Warrington, PA, USA). Samples of 65 nm thick sections were stained with lead citrate and uranyl acetate. The stained samples were imaged using TEM (Philips CM200; Field Emission Instruments, Hillsboro, OR, USA) equipped with an XR41B CCD camera (Advanced Microscopy Techniques, Woburn, MA, USA).

### 2.9. Statistical Analysis

The mean ± standard error of the mean was used to present the final result. Statistical analysis was conducted using nonparametric analysis of variance, followed by Kruskal-Wallis’s multiple comparison test for multiple groups using GraphPad Prism version 7.03 (GraphPad Software Inc., San Diego, CA, USA). Statistical significance was determined if *p* values were <0.05.

## 3. Results

### 3.1. Bavachin and Corylifol a Delay the Pathological Progression of Muscle Atrophy in DEX-Treated Mice

**Figure 1:**
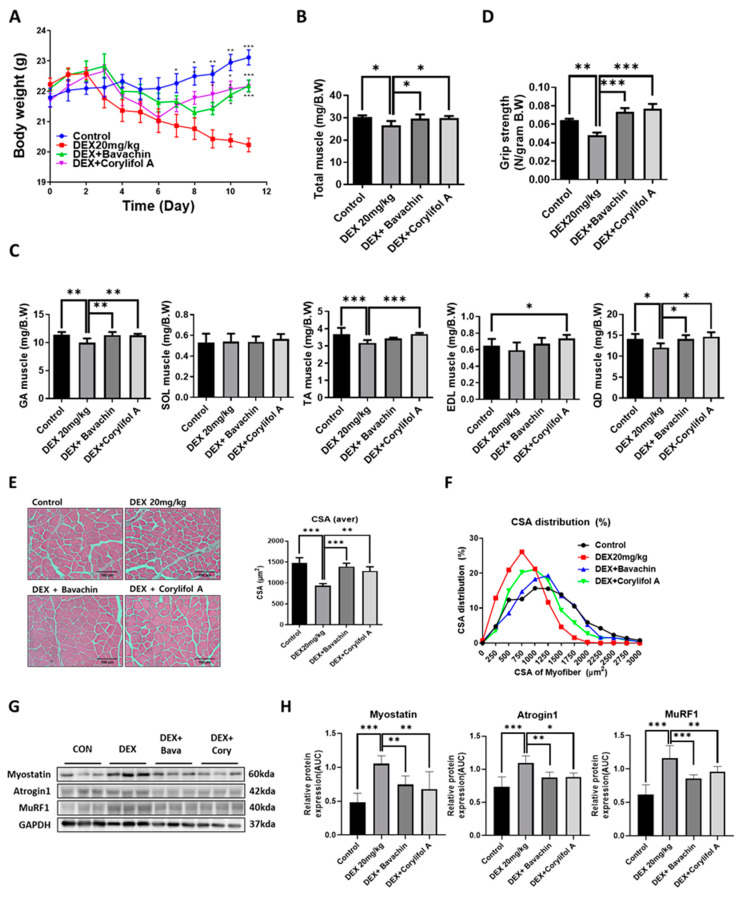
Bavachin (10 mg/kg) or corylifol A (10 mg/kg) was orally administered for 12 d, starting two days prior to DEX administration. (**A**) Body weight changes in mice and (**B**) total muscle weight. (**C**) Muscle weights of the gastrocnemius (GA), soleus (SOL), tibialis anterior (TA), extensor digitorum longus (EDL), and quadriceps femoris (QD) muscles. (**D**) Grip strength. (**E**) Representative images of the cross-sectional area (CSA) in the GA muscle tissue at 200× magnification. Average CSA of muscle fibers. (**F**) CSA distribution of the muscle fibers. CSA data were measured using the ImageJ software. Protein expression levels in GA muscles were determined via western blotting. (**G**) Representative images of the atrophic muscle factors. (**H**) Protein expression levels of the atrophic muscle factors. All data are represented as the mean ± standard error of the mean (S.E.M)., *n* = 6/group; * *p* < 0.05, ** *p* < 0.01, *** *p* < 0.001; nonparametric analysis of variance, followed by Kruskal-Wallis test.

To identify the components of PCS that can improve mitochondrial function, we screened for measured COX activity in C2C12 myotubes. Among the PCS components, bavachin and corylifol A significantly restored the decreased COX activity induced by DEX (Figure S1). Based on these data, we first investigated the potential effects of bavachin and corylifol A on the pathological progression of muscle atrophy in DEX-treated mice. Body weight decreased gradually with DEX but increased on days six and eight, after the administration of corylifol A and bavachin, respectively, and were identical at the end of administration (Figure 1A). Additionally, DEX administration reduced the total muscle mass compared to that of the control. In particular, DEX was highly effective in inducing muscle atrophy in the GA, TA, and QD muscle types. In contrast, the atrophic effect was blunted. Muscle mass was recovered by treatment with bavachin or corylifol A (Figure 1B,C). Consistently, grip strength increased in both groups of mice compared to that in the DEX group, and its level was comparable to that of the control group (Figure 1D). In line with the grip strength data, the CSA of myofibers was significantly decreased by DEX compared to that of the CON group. Conversely, the CSA was significantly increased after bavachin or corylifol A administration (Figure 1E,F). While the pattern of myofiber size distribution in the DEX group was dominant in the range of 250–1250 μm^2^, that of the 1500–3000 μm^2^ myofibers was drastically decreased. The administration of bavachin or corylifol A reversed this distribution pattern to that of the control group (Figure 1F). Next, we determined the protein expression levels of myostatin, atrogin-1, and MuRF 1, which are implicated in muscle atrophy in the GA muscle. DEX enhanced the protein expression levels of myostatin, atrogin-1, and MuRF 1 compared to those of the control group (Figure 1G,H). In contrast, this effect was significantly inhibited by bavachin or corylifol A treatment (Figure 1G,H).

### 3.2. Bavachin and Corylifol a Enhance the Muscle Function in Mice with Diabetes-Induced Muscle Atrophy

**Figure 2:**
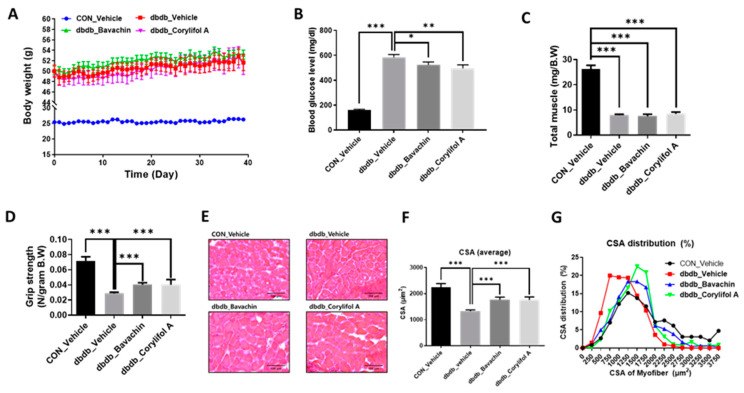
Bavachin and corylifol A increase the muscle strength in diabetic (db/db) mice. Twelve week old male db/db mice were administered bavachin (10 mg/kg) or corylifol A (10 mg/kg) for 40 d. (**A**) Body weight changes in mice, and (**B**) non-fasting blood glucose levels. (**C**) Total muscle weight. (**D**) Grip strength. (**E**) Representative images of CSA of GA muscle tissue at 200× magnification. (**F**) Average CSA of muscle fibers. (**G**) CSA distribution of muscle fibers. CSA data were measured using the ImageJ software. All data are represented as the mean ± S.E.M., *n* = 6–7/group; * *p* < 0.05, ** *p* < 0.01, *** *p* < 0.001; nonparametric analysis of variance, followed by Kruskal-Wallis test.

Since T2D is known to induce muscle atrophy, we investigated the effects of bavachin and corylifol A on the muscles in db/db mice; db/db mice showed an increase in body weight and blood glucose levels compared to those in the control mice (Figure 2A,B). Administration of bavachin or corylifol A did not change the body weight, but significantly lowered the blood glucose levels in db/db mice (Figure 2A,B). In particular, this reduction was more effective with corylifol A (Figure 2B). The total muscle weight of db/db mice was significantly lower than that of the control mice and was not increased by the administration of bavachin or corylifol A (Figure 2C). In addition, grip strength showed a reduction of up to 60% in db/db mice compared to that of the control group (Figure 2D). The administration of bavachin or corylifol A significantly enhanced the grip strength in db/db mice as shown in Figure 1D. In line with the grip strength data, CSA was decreased by approximately 40% in db/db mice compared to that of the control mice (Figure 2E,F). Conversely, this reduction was significantly reversed by the administration of bavachin or corylifol A (Figure 2E,F). While the myofiber size distribution pattern in db/db mice was dominant in the range of 250–1250 μm^2^, 1750–3750 μm^2^ myofibers were significantly decreased (Figure 2G). In contrast, the administration of bavachin or corylifol A shifted this distribution pattern to large size myofibers (Figure 2G).

### 3.3. Bavachin and Corylifol a Inhibit Muscle Atrophy by Suppressing the Inflammatory Cytokine Expression Levels in db/db Mice

**Figure 3:**
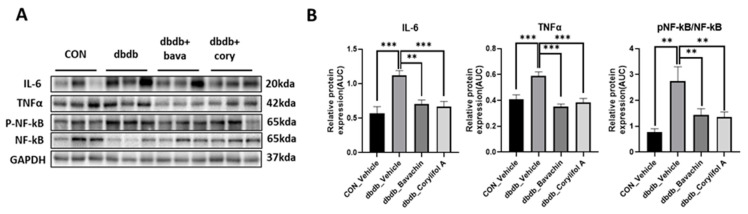
Bavachin and corylifol A suppress pro-inflammatory cytokine expression levels in db/db mice. Twelve week old male db/db mice were administered bavachin (10 mg/kg) or corylifol A (10 mg/kg) for 40 d. Protein expression levels in the GA muscle were determined using western blotting. (**A**) Representative images of western blotting. (**B**) Protein expression levels of inflammatory cytokines. All data are represented as the mean ± S.E.M., *n* = 6/group; ** *p* < 0.01, *** *p* < 0.001; nonparametric analysis of variance, followed by Kruskal-Wallis test.

**Figure 4:**
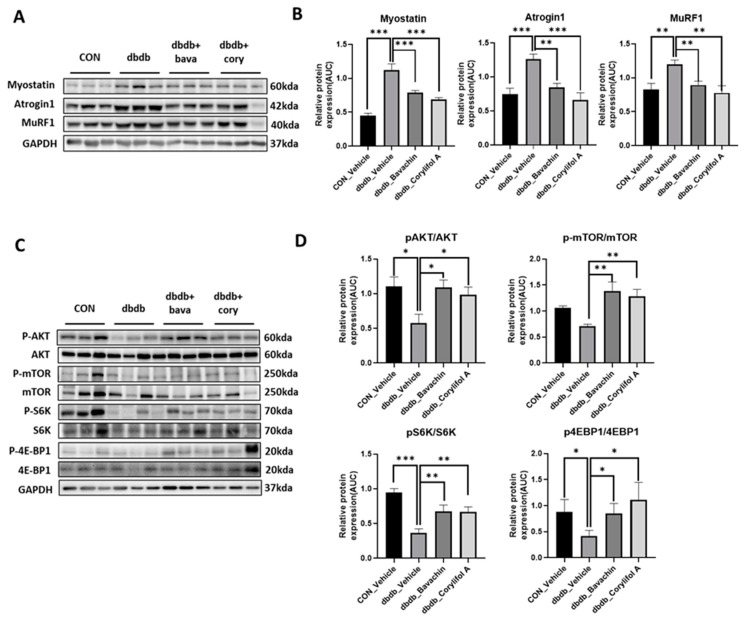
Bavachin and corylifol A decrease muscle atrophy in db/db mice. Twelve week old male db/db mice were administered bavachin (10 mg/kg) or corylifol A (10 mg/kg) for 40 d. Protein expression levels in GA muscles were measured using western blotting. (**A**) Representative images of the atrophic muscle factors. (**B**) Protein expression levels of the atrophic muscle factors. (**C**) Representative images of the AKT signaling pathway. (**D**) Protein expression levels in the AKT signaling pathway. All data are represented as the mean ± S.E.M., *n* = 6/group; * *p* < 0.05, ** *p* < 0.01, *** *p* < 0.001; nonparametric analysis of variance, followed by Kruskal-Wallis test.

Chronic inflammation is characteristic of T2D, and inflammation is a risk factor for inducing muscle atrophy by upregulating the levels of muscle atrophy-related factors, such as atrogin-1 and MuRF-1 [6,29]. Therefore, we first examined the protein expression levels of TNF-α and IL-6, and NF-κB signaling pathway activity associated with the expression of inflammatory genes, such as cytokines [30,31]. NF-κB activity was increased in db/db mice, which, in turn, resulted in increased TNF-α and IL-6 levels compared to those of the control mice (Figure 3A,B). In contrast, bavachin or corylifol A administration significantly suppressed the levels of NF-κB phosphorylation and its downstream target proteins, such as TNF-α and IL-6 (Figure 3A,B). This effect was comparable for both administrations, as shown in Figure 3. Next, we investigated whether the ubiquitin–proteasome pathway was activated in db/db mice. As expected, the levels of muscle-specific ubiquitin ligases, atrogin-1 and MuRF-1, were significantly increased with elevated myostatin expression levels in db/db mice (Figure 4A,B). In contrast, bavachin or corylifol A administration reversed this effect (Figure 4A,B). Moreover, AKT-mediated protein synthesis signaling pathway was significantly reduced in db/db mice (Figure 4C,D). However, bavachin or corylifol A administration significantly increased the phosphorylation of AKT and mTOR/S6K/4EBP1 (Figure 4C,D).

### 3.4. Bavachin and Corylifol a Attenuate Muscle Fiber and Mitochondrial Damage in Skeletal Muscles of db/db Mice

**Figure 5:**
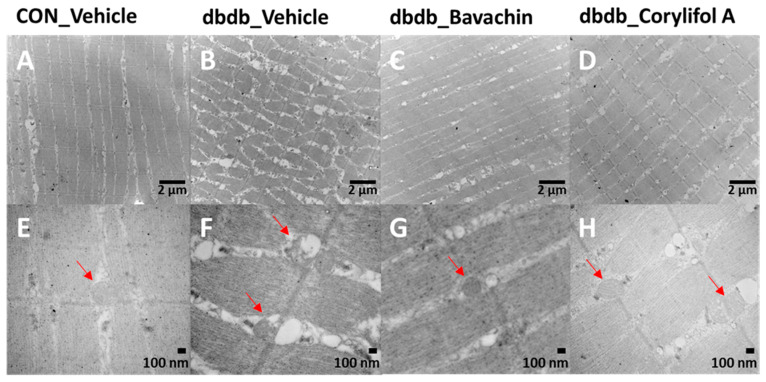
Bavachin and corylifol A improve the organization of muscle fibers and attenuate mitochondrial damage in skeletal muscles of db/db mice. Twelve week old male db/db mice were administered bavachin (10 mg/kg) or corylifol A (10 mg/kg) for 40 d. Transmission electron microscopy (TEM) of the GA muscle. (**A**,**E**) Control + vehicle, (**B**,**F**) db/db + vehicle, (**C**,**G**) db/db + bavachin, and (**D**,**H**) db/db + corylifol A groups. Red arrowheads indicate mitochondria. Images (**A**–**D**) were captured at 8000× magnification, and (**E**,**F**) at 20,000× magnification.

TEM showed damaged or swollen mitochondria, disordered arrangement of muscle fibers, and irregular fiber structure in db/db mice compared with the control mice (Figure 5). However, bavachin or corylifol A administration significantly changed the ultrastructure of the skeletal muscle, as shown in Figure 5, with an ordered arrangement of muscle fibers and many continuous and elongated mitochondria.

### 3.5. Bavachin and Corylifol a Induce the Muscle Fiber Switch from Fast-Type Glycolytic Fibers to Slow-Type Oxidative Fibers in db/db Mice

**Figure 6:**
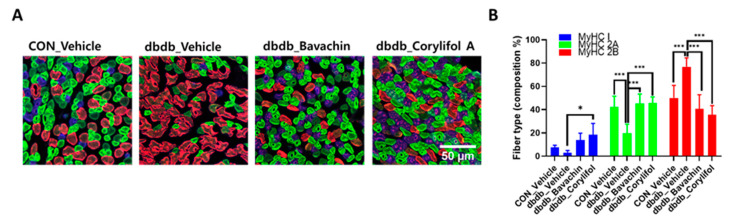

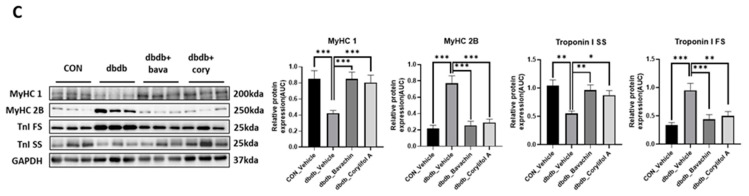
Bavachin and corylifol A induce the conversion of fast muscle fibers to slow muscle fibers in db/db mice. Twelve week old male db/db mice were administered bavachin (10 mg/kg) or corylifol A (10 mg/kg) for 40 d. (**A**) Representative images of MyHC immunofluorescence in GA muscle. Images were triple labeled for MyHC 1 (blue, type 1 fibers), MyHC 2A (green, type 2A fibers), and MyHC 2B (red, type 2B fibers). Scale bar indicates 50 µm. Images were captured at 600× magnification. (**B**) Proportion of MyHC isoform-specific fibers. Protein expression levels in the GA muscles were determined via western blotting. (**C**) Protein expression levels of MyHC 1, MyHC 2B, troponin I fast skeletal muscle (TnI-FS) and troponin I slow skeletal muscle (TnI-SS). All data are represented as the mean ± S.E.M., *n* = 6/group; * *p* < 0.05, ** *p* < 0.01, *** *p* < 0.001; nonparametric analysis of variance, followed by Kruskal-Wallis test.

To further determine whether bavachin or corylifol A administration induces the conversion of glycolytic fibers into oxidative fibers in db/db mice, we analyzed GA muscle cryosections for myosin heavy chain (MyHC) isoforms type I, 2A, and 2B via multicolor staining (Figure 6). In db/db mice, while MyHC I and 2A expression levels were decreased, MyHC 2B expression levels were significantly increased compared with those in the control mice (Figure 6A,B). In contrast, bavachin or corylifol A administration increased MyHC I and 2A expression to levels similar to those in the control mice (Figure 6A,B). Specifically, bavachin and corylifol A administration suppressed MyHC 2B expression in db/db mice (Figure 6A,B). Consistently, the protein expression levels of the MyHC isoform in GA muscle showed a similar trend to the staining pattern. Specifically, while the protein expression levels of MyHC I were significantly increased, those of MyHC 2B were decreased by bavachin or corylifol A treatment (Figure 6C). To further confirm the fiber-type transition, we determined the protein expression levels of troponin I fast skeletal muscle (TnI-FS) and troponin I slow skeletal muscle (TnI-SS) in GA muscles representing fast and slow fiber types, respectively. In db/db mice, TnI-FS protein expression levels were increased, but those of TnI-SS were decreased compared with those in the control mice (Figure 6C). This change in protein expression levels was reversed by the administration of bavachin or corylifol A (Figure 6C).

### 3.6. Bavachin and Corylifol a Improve the Mitochondrial Dynamics via the AMPKα Signaling Pathway in db/db Mice

**Figure 7:**
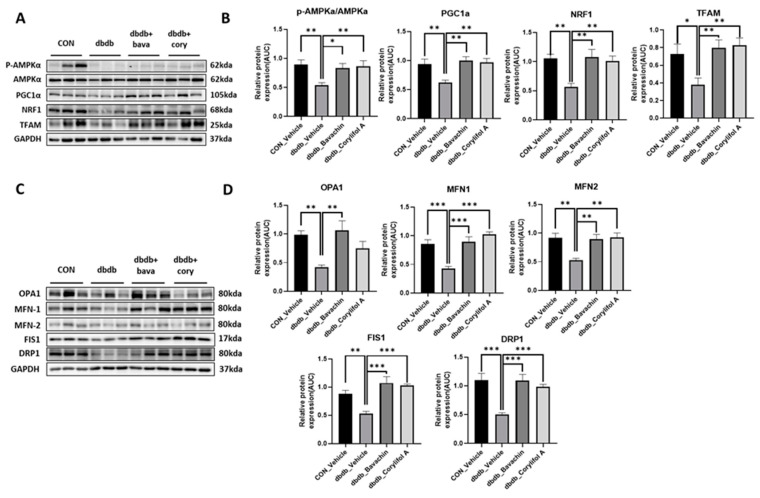
Bavachin and corylifol A induce mitochondrial dynamics in the skeletal muscle via the AMP-activated protein kinase (AMPK) signaling pathway. Twelve week old male db/db mice were administered bavachin (10 mg/kg) or corylifol A (10 mg/kg) for 40 d. Protein expression levels in the GA muscle were measured via western blotting. (**A**) Representative images of mitochondrial biogenesis. (**B**) Protein expression levels of mitochondrial biogenesis. (**C**) Representative images of fusion and fission factors. (**D**) Protein expression levels of fusion and fission factors. All data are represented as the mean ± S.E.M., *n* = 6/group; * *p* < 0.05, ** *p* < 0.01, *** *p* < 0.001; nonparametric analysis of variance, followed by Kruskal-Wallis test.

Mitochondrial biogenesis and dynamics are critical for mitochondrial function [32]. In addition, changes in muscle fiber composition induced by bavachin and corylifol A are associated with mitochondrial number and function [33]. We investigated mitochondrial biogenesis and dynamics. PGC-1α, NRF1, and TFAM expression levels were significantly decreased in db/db mice as a result of reduced AMPKα phosphorylation (Figure 7A,B). Bavachin and corylifol A increased AMPKα phosphorylation and the expression levels of PGC-1α, NRF1, and TFAM (Figure 7A,B). Expression levels displayed no difference between the bavachin and corylifol A groups (Figure 7A,B). Accurate regulation of mitochondrial morphology is important for a smooth supply of intracellular energy, and quantitative and qualitative regulation of mitochondrial morphology-regulating proteins is required to achieve an appropriate balance between mitochondrial fission and fusion processes [34,35,36]. Expression levels of all fusion (OPA1, MFN1, and MFN2) and fission (FIS1 and DRP1)-related proteins were decreased in db/db mice compared with those in the control mice (Figure 7C,D). Similarly, the expression levels of all fusion (OPA1, MFN1, and MFN2) and fission (FIS1 and DRP1)-related proteins were decreased in db/db mice compared to those in the control mice (Figure 7C,D). However, this decrease in expression, except for that of OPA1, in the case of corylifol A administration, was significantly increased by both treatments (Figure 7C,D).

### 3.7. Bavachin and Corylifol a Improve the Mitochondrial Quality by Upregulating Mitophagy in Muscles

**Figure 8:**
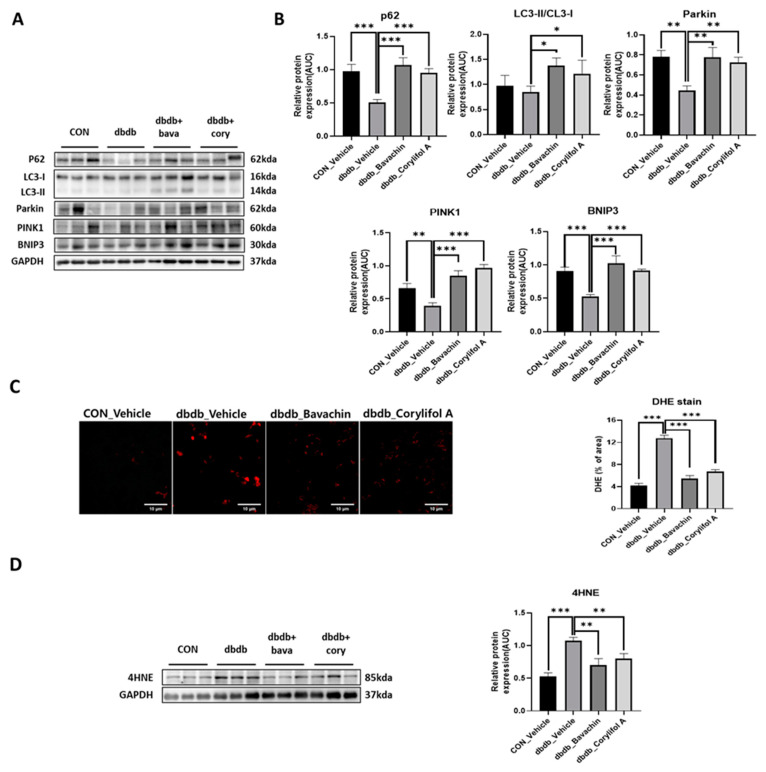
Bavachin and corylifol A promote mitophagy and improve the mitochondrial homeostasis. Twelve week old male db/db mice were administered bavachin (10 mg/kg) or corylifol A (10 mg/kg/day) for 40 d. Protein expression levels in the GA muscle were measured via western blotting. (**A**) Representative images of mitophagy factors. (**B**) Protein expression levels of mitophagy factors. (**C**) Dihydroethidium (DHE) staining. Images were captured at 600× magnification. (**D**) Protein expression levels of 4-hydroxynonenal (4HNE). All data are represented as the mean ± S.E.M., *n* = 6/group; * *p* < 0.05, ** *p* < 0.01, *** *p* < 0.001; nonparametric analysis of variance, followed by Kruskal-Wallis test.

Mitophagy can cause aging and damage to mitochondria, which are important for the control of mitochondrial quality [37]. We analyzed the expression of proteins involved in autophagic processes. LC3 II is a marker of autophagosome formation, whereas p62 is a marker of autophagolysosome degradation. We found a significant decrease in p62 levels in db/db mice, whereas the expression levels of LC3 II were not changed in db/db mice (Figure 8A,B). Bavachin increased the expression levels of p62 and LC3 II, and corylifol A significantly increased the expression levels of p62 compared with those in db/db mice (Figure 8A,B). PINK1/Parkin-mediated pathway is one of the major mitophagy pathways in mammals [38]. Parkin and PINK1 protein expression levels were reduced in db/db mice. These effects were reversed by the administration of bavachin or corylifol A (Figure 8A,B). Mitophagy can also occur via parkin independent pathways. Levels of BNIP3, a biomarker of the parkin independent pathway, were decreased in db/db mice, but increased by the administration of bavachin or corylifol A (Figure 8A,B). These results suggest that both mediated mitophagy pathways were activated by bavachin and corylifol A. Consistently, ROS production levels from failed mitochondrial quality control were significantly elevated in db/db mice, as confirmed by bright positive areas (Figure 8C) and increased 4HNE levels (Figure 8D). Conversely, bavachin and corylifol A administration decreased DHE fluorescence brightness and 4HNE protein levels (Figure 8C,D).

## 4. Discussion

Mitochondrial quality control plays an important role in the maintenance of muscle mass. High production of ROS caused by mitochondrial dysfunction can induce oxidative stress, resulting in tissue damage [39]. Therefore, the enhancement of mitochondrial biogenesis and function can be a strategy for increasing muscle function and muscle mass.

We previously reported that PCS attenuates muscle atrophy in DEX-induced muscle atrophy models by suppressing oxidative stress and inflammation [25]. In the current study, we identified bavachin and corylifol A as agents for enhancing mitochondrial function and used them to investigate their potential in vivo effects in both DEX-induced muscle atrophic mice and db/db mice. Our findings indicated that bavachin and corylifol A faithfully recapitulated our previous observations with PCS in DEX-induced muscle atrophic mice. In diabetic mice, these two components improved muscle strength by suppressing inflammation-mediated muscle atrophy and inducing a muscle fiber switch from fast-type glycolytic fibers (type 2B) to slow-type oxidative fibers (type I, 2A). In addition, they increased mitochondrial biogenesis and dynamic factor expression (OPA1, MFN1/2, FIS1, and DRP1) via the AMPKα–PGC-1α signaling pathway, targeting NRF1 and TFAM. Moreover, bavachin and corylifol A in db/db mice improved mitochondrial quality by upregulating mitophagy factors (p62, Parkin, PINK1 and BNIP3) in the muscle, resulting in the reduction of oxidative stress in diabetic mice. These data suggest that bavachin and corylifol A are the main effectors among the components of PCS and have a potential therapeutic effect on muscle atrophy.

T2D is characterized by elevated levels of circulating, pro-inflammatory cytokines, such as TNFα and IL-6 [40]. Inflammatory factors can directly activate a series of downstream signaling pathways via their receptors, thereby suppressing muscle protein synthesis, over-activating proteolysis, and resulting in skeletal muscle atrophy [41,42]. Consistently, our data also showed increased NF-κB-mediated inflammatory cytokine expression, such as IL-6 and TNFα, and activation of the ubiquitin proteasome system, as demonstrated by an increase in atrogin-1 and MuRF-1 expression, followed by the induction of muscle atrophy in db/db mice. In contrast, while this elevation was significantly suppressed, the Akt–mTOR–S6K–4E-BP1 signaling pathway involved in protein synthesis was simultaneously activated by bavachin and corylifol A. However, there was no increase in total muscle mass in db/db mice, unlike that in DEX-induced muscle atrophic mice. CSA is often used to assess muscle regeneration efficacy and is well correlated with muscle strength [43,44,45]. Consistent with the literature, in both mouse models our data showed that grip strength was significantly increased with an increase in CSA by bavachin and corylifol A administration. Taken together, these data suggest that muscle mass is not a more decisive factor than muscle function in grip strength.

Mitochondrial content and function are important factors in skeletal muscle to maintain muscle strength and determine muscle fiber types [28,46]. Type I and 2A fibers are slower fibers, because of their higher mitochondrial content and activity, than type 2X fibers, which are characteristic of oxidative metabolism [47]. In obese individuals with diabetes, the activity of slow muscles is reduced compared to that of fast muscles, similar to the effect of a shift in fiber type from type I and 2A fibers to type 2X fibers. [48,49]. Similarly, our current data showed that atrophy in the GA muscle of db/db mice increased with a fiber-type shift from type 1 to type 2B. However, bavachin and corylifol A increased MyHC 1 and 2A levels, indicating slow-type fibers, but decreased MyHC 2B levels, indicating fast-type fibers, as shown by immunofluorescence and immunoblot data (decrease in TnI-FS expression and an increase in TnI-SS expression). In addition, ultrastructural analysis of muscle fibers and mitochondria by TEM revealed damaged mitochondria and impaired order of muscle fibers in db/db mice; however, this abnormal structure was reversed by either administration. Taken together, these data indicate that the major target organelles of bavachin and corylifol A are the mitochondria. These results demonstrate that the components of PCS may enhance mitochondrial activity, restore the mitochondrial respiratory chain, and improve energy metabolism in the skeletal muscle of T2D mice.

Mitochondria have a complex interconnected reticulum, and this structure is maintained by remodeling processes collectively known as mitochondrial quality control [10]. Mitochondrial biogenesis, fission, fusion, and mitophagy are key events in mitochondrial quality control [10]. PGC-1α is an expert regulator of mitochondrial biogenesis, and its expression is induced by exercise and chemical activation of AMPKα in the skeletal muscle [11]. AMPKα regulates transcription factors, such as NRF-1, CREB, MEF2, and PGC-1α by phosphorylation [50]. AMPKα regulates mitochondrial biogenesis through a PGC-1α-dependent mechanism [11,51]. Bavachin and corylifol A increased the phosphorylation of AMPKα, which was decreased in db/db mice, thereby increasing PGC-1α, NRF-1, and TFAM protein expression. These data indicate that bavachin and corylifol A may directly induce mitochondrial biogenesis via the AMPKα–PGC-1α signaling pathway targeting NRF1 and TFAM. Mitochondrial quality control mechanisms maintain mitochondrial function through selective fusion, fission, and mitophagy [52]. While OPA1 interacts with the mammalian mitofusins MFN1 and 2 to promote mitochondrial fusion [53,54,55], DRP1, and FIS1 promote mitochondrial fission [56]. In diabetic mice, MFN1/2, OPA1, DRP1, and FIS1 protein expression was significantly decreased compared with those in control mice. In contrast, bavachin and corylifol A administration reversed these effects.

Mitophagy is an essential cellular event that maintains a healthy mitochondrial population by removing damaged mitochondria [57]. The key regulators of mitophagy are PINK1, ubiquitin ligase PARKIN, ubiquitin, and p62 [58]. PINK1 expression is significantly lower in the skeletal muscles of patients with T2DM than that in control subjects [59]. Moreover, several studies have shown that a reduction in mitophagy contributes to mitochondrial dysfunction not only in aged mice and patients with mitochondrial myopathy, but also in peripheral blood mononuclear cells from patients with T2D [60,61]. This report suggested that DM may inhibit appropriate mitophagy, resulting in the accumulation of damaged mitochondria in the skeletal muscle. Similarly, our data showed that PINK1 expression, an indispensable mediator of mitophagy, was significantly decreased in diabetic mice. However, bavachin and corylifol A administration significantly elevated all mediators of mitophagy, suggesting that the impaired mitophagy system was restored in diabetic mice, as evidenced by a reduction in ROS production.

In conclusion, we found that bavachin and corylifol A improved muscle strength and induced a muscle fiber switch from fast-type glycolytic fibers to slow-type oxidative fibers in diabetic mice via three main mechanisms: (1) a decrease in protein degradation and increase in protein synthesis by attenuating inflammation; (2) improving mitochondrial function by regulating mitochondrial biogenesis and dynamics via the AMPKα–PGC-1α signaling pathway targeting NRF1 and TFAM; (3) improvement in mitochondrial quality by upregulating the expression levels of mitophagy factors (p62, Parkin, PINK1, and BNIP3), resulting in the reduction of oxidative stress. These findings suggest that bavachin and corylifol A exert potential therapeutic effects on muscle atrophy in diabetic mice.

## Data Availability

The data presented in this study are available in the article and Supplementary Materials.

## Supplementary Materials

The following supporting information can be downloaded at: https://www.mdpi.com/article/10.3390/antiox12010137/s1, Figure S1: Components of *Psoralea corylifolia* L. seed increase the cytochrome C oxidase activity in Dexamethasone (DEX)-treated C2C12 myotubes.
