# Supplementary material for: Bavachin and Corylifol A Improve Muscle Atrophy by Enhancing Mitochondria Quality Control in Type 2 Diabetic Mice
Source: Antioxidants (Basel). 2023 Jan 6;12(1):137. doi: 10.3390/antiox12010137 (PMC9855061; doi:10.3390/antiox12010137)
Supplement: Supplementary file 1 [file antioxidants-12-00137-s001.zip › antioxidants-2081674-SI.pdf]

Supplementary Material

Bavachin and Corylifol A Improve Muscle Atrophy by Enhancing Mitochondria Quality Control in Type 2 Diabetic Mice

MyeongHoon Yeon [1], Eunhui Seo [1,2], Jong-Han Lee [3,*] and Hee-Sook Jun [1,2,4,*]

1 College of Pharmacy and Gachon Institute of Pharmaceutical Sciences, Gachon University, Incheon 21936, Republic of Korea
2 Lee Gil Ya Cancer and Diabetes Institute, Gachon University, Incheon 21999, Republic of Korea
3 Department of Marine Bio and Medical Science, Hanseo University, Seosan-si, 31962, Republic of Korea
4 Gachon Medical Research Institute, Gil Hospital, Incheon 21565, Republic of Korea
* Correspondence: Jong Han Lee, Department of Marine Bio and Medical Science, Hanseo University, Seosan-si, Chungcheongnam-do, 31962, Republic of Korea, E-mail: jhleecw3@hanseo.ac.kr; Hee-Sook Jun, College of Pharmacy, Gachon University, Incheon 21936, Republic of Korea, Email: hsjun@gachon.ac.kr

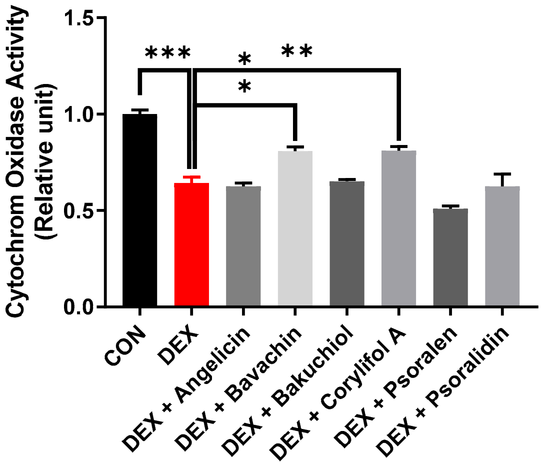

Figure S1. Components of *Psoralea corylifolia* L. seed increase the cytochrome C oxidase activity in Dexamethasone (DEX)-treated C2C12 myotubes. C2C12 myotubes were incubated with 1 μM DEX, with or without 10 μM *P. corylifolia* L. seed components (angelicin, bavachin, bakuchiol, corylifol A, psoalen, and psoralidin), for 24 h. Data are represented as the mean ± standard error of the mean (S.E.M.). n = 3. **$p < 0.01$, ***$p < 0.001$ vs. CON, one-way analysis of variance (ANOVA; Tukey).

Materials and Methods

Measurement of Cytochrome C Oxidase (COX) Activity

Mitochondrial respiratory chain complex IV (COX) activity was determined using a COX assay kit (ab239711; Abcam). C2C12 myotubes were incubated with 1 μM DEX with or without 10 μM PCS components (angelicin, bavachin, bakuchiol, corylifol A, psoalen, and psoralidin) for 24 h. Briefly, a suitable volume of mitochondrial solution containing 20 μg of protein was added to the assay buffer in each well of a 96-well plate. After adding the reaction solution, changes in optical density values at 550 nm were recorded immediately using a kinetic program for 45 min at 1-min intervals.
